# Comparative Study on ZnO Monolayer Doped with Al, Ga and In Atoms as Transparent Electrodes

**DOI:** 10.3390/ma10070703

**Published:** 2017-06-26

**Authors:** Dan Sun, Changlong Tan, Xiaohua Tian, Yuewu Huang

**Affiliations:** 1 College of Applied Science, Harbin University of Science and Technology, Harbin 150080, China; wyysundan@gmail.com (D.S.); xiaohuatian@hrbust.edu.cn (X.T.); 2 School of Materials Science and Engineering, Harbin Institute of Technology, Harbin 150001, China; wuyuehuang@gmail.com

**Keywords:** ZnO monolayer, electronic structure, optical properties, transport proprieties, first-principle

## Abstract

Transparent anodes are indispensable components for optoelectronic devices. Two-dimensional (2D) materials are attracting increasing research interest due to their unique properties and promising applications. In order to design novel transparent anodes, we investigated the electronic, optical, and electrical properties of 2D ZnO monolayers doped with Al, Ga, and In using the first-principles calculation in combination with the Boltzmann transport theory. When the doping concentration of Al, Ga, and In is less than 12.5 wt %, we find that the average transmittance reaches up to 99% in the visible and UV regions. Moreover, the electrical conductivity is enhanced for the Al, Ga, and In doped systems compared to that of the pristine ZnO monolayer. In particular, a good electrical conductivity with a significant improvement for the In doped ZnO monolayer is achieved compared to Al and Ga doping at the 6.25 wt % level. These results suggest that the ZnO monolayer based materials, and in particular the In doped ZnO monolayer, are promising transparent anodes for nanoscale electronic and optoelectronic applications.

## 1. Introduction

Zinc oxide (ZnO) has emerged as a promising semiconductor material because of its particular electric and optical properties. It has a wide band gap (3.37 eV) at room temperature and a large excitation binding energy [1,2]. Such properties make it well suited for a variety of applications such as those in transparent electronics, ultraviolet (UV) light emitters, and piezoelectric devices [3,4,5]. Therefore, research on ZnO has become a hot topic in the optoelectronic fields.

The doping technique is an efficient approach for tuning the physical properties and improving the optical and electrical properties of the ZnO materials. Al doped ZnO has been extensively explored as transparent conducting films with high electrical conductivity [6]. Ng et al. [7] found a high resistivity and a significant transparency around 90% in the 370–800 nm wavelength range for Ga and Al doped ZnO by using the sol-gel spin coating technique. Hsu et al. [8] noted an increase in gap energy after doping with Ga and In, but a reduced band gap for Al doped ZnO compared to pristine ZnO. Meanwhile, Kuprenaite et al. [9] showed that doping ZnO with Al, In, and Ga enhances the transmittance of the materials and decreases their resistivity at low dopant concentrations. These results show that doping ZnO with group III elements makes it a good transparent conducting electrode for optoelectronic device applications.

Up to now, most of the existing research on group Ш element doped ZnO focused on bulk and thin films forms [10,11,12,13]. Recently, 2D materials are attracting increasing research interest due to their unique properties, such as the electron confinement of 2D materials without interlayer interactions, the maximum mechanical flexibility, high optical transparency, large lateral size, and ultrathin thickness. They have obtained large interest for potential applications in the fabrication of highly flexible and transparent optoelectronic devices [14]. To the best of our knowledge, there are few investigations on 2D ZnO monolayers doped with group Ш elements [15]. The discovery of 2D graphene has led to the intense interest in other potential 2D materials with novel properties [16]. As the allotrope of the superior bulk ZnO, the 2D ZnO sheet logically triggers scientists’ interest [17,18,19,20,21,22,23,24,25]. Freeman et al. [26] first theorized that free-standing thin films of wurtzite ZnO are less stable than a phase based on 2D ZnO. Tusche et al. [27] observed two-monolayer-thick ZnO (0001) films grown on Ag substrates through surface X-ray diffraction and scanning tunneling microscopy. Our research and previous studies show that the 2D ZnO monolayer exhibits distinct properties when compared with the bulk and film ZnO due to the quantum confinement effect [28]. Thus, it is desirable to explore the potential of group Ш element doped ZnO monolayers as novel transparent electrode materials and furthermore propose the choice of the dopant from these candidates to obtain a transparent conducting oxide (TCO) with good properties.

Good TCOs should have a large transmittance in the visible region and high electrical conductivity. Therefore, the present study is dedicated to investigating the electronic, optical, and electrical properties of group Ш element doped ZnO monolayers by means of the first principles calculation in combination with the Boltzmann transport equation. We have found that the average transmittance reaches up to 99% in the visible and UV regions when the Al, Ga, and In doping concentration is less than 12.5 wt %. Meanwhile, the electrical conductivity of In doped at 6.25 wt % concentration is larger than that of the Al and Ga doped ZnO monolayers. Our results may provide guidance for designing novel transparent electrodes based on ZnO monolayers.

## 2. Calculation Models and Methods

All of the calculations were performed with the CASTEP code 8.0 and the ultrasoft pseudopotentials method, which were based on the density functional theory [29]. All of the structures were treated with periodic boundary conditions. The supercell was large enough to ensure a vacuum spacing greater than 15 Å. The exchange correlation functional was described by the generalized gradient approximation (GGA) in the form of Perdew-Burke-Ernzerhof (PBE) [30]. The structure optimization and the ground state calculations were performed with a cut-off energy of 400 eV for the basis set and a 4 × 4 × 1 Monkhorst-Pack grid for the Brillouin zone (BZ) sampling [31]. The convergence of the total energy was considered to be achieved when two iterated steps with an energy difference less than 10^−6^ eV occurred. The structural relaxation was performed until the forces on each atom were smaller than 0.02 eV Å^−1^.

The standard density functional theory (DFT ) underestimates the band gap of many transition metal oxides. Therefore, to obtain a more accurate representation of the electronic structure of ZnO, we adopt the DFT + *U_d_* + *U_p_* method to investigate all of the structures, which was also used in our previous study [28]. The *U_d_* value for Zn 3*d* and the *U_p_* value of O 2*p* were considered as 10 eV and 7 eV, respectively. The calculated band gap (3.37 eV) is in good agreement with the experimental measurements [5]. Meanwhile, the lattice parameters are a = 3.313 Å and c = 5.329 Å, which are close to the experimental values of a = 3.249 Å and c = 5.206 Å [32].

In order to describe all of the optical properties of the structures, the dielectric function is expressed as $\varepsilon \left(\omega \right)=\varepsilon_{1}\left(\omega \right)+i\varepsilon_{2}\left(\omega \right)$, which is mainly contributed from the electronic structures. $\varepsilon_{1}$ and $\varepsilon_{2}$ are the real part and the imaginary part of the dielectric function, respectively. $\varepsilon_{2}$ is calculated as follows [33]

(1) $$\varepsilon_{2}=\frac{4\pi^{2}}{m^{2}\omega^{2}}\underset{V,C}{\sum }\int_{BZ}d^{3}k\frac{2}{2\pi }{\left|e\cdot M_{CV}\left(K\right)\right|}^{2}\times \delta \left[E_{C}\left(K\right)-E_{V}\left(K\right)-\hbar \omega \right],$$

where ω is the frequency of light, C is the conduction band, V is the valence band, BZ is the first Brillouin Zone, K is the reciprocal lattice vector, and M is the dipole matrix.

$\varepsilon_{1}$ is obtained from $\varepsilon_{2}$ by using the Kramer-Kronig transformation [34].

(2) $$\varepsilon_{1}=1+\frac{8\pi^{2}}{m^{2}\omega^{2}}\underset{V,C}{\sum }\int^{\text{​}}d^{3}k\frac{2}{2\pi }\times \frac{{\left|e\cdot M_{CV}\left(K\right)\right|}^{2}}{E_{C}\left(K\right)-E_{V}\left(K\right)}\times \frac{\hbar^{3}}{\left[E_{C}\left(K\right)-E_{V}\left(K\right)-\hbar^{2}\omega^{2}\right]},$$

The derivation of the absorption coefficient I(ω) and the reflection coefficient R(ω) based on the dispersion relations and transition probability can be described as follows [35,36,37].

(3) $$I\left(\omega \right)=\sqrt{2}\omega {\left[\sqrt{\varepsilon_{1}\left(\omega \right)+\varepsilon_{2}\left(\omega \right)}-\varepsilon_{1}\left(\omega \right)\right]}^{1/2},$$

(4) $$R\left(\omega \right)=\frac{{\left(n-1\right)}^{2}+k^{2}}{{\left(n+1\right)}^{2}+k^{2}},$$

where n stands for the refractive index.

To calculate the electrical properties of the pristine and doped ZnO monolayer, the calculated band structure data from DFT is fitted into the Boltzmann package that is based on the semi-classic Boltzmann theory and the rigid band approach [38,39]. It follows from these approaches that the dependence of the conductivity on the transport distribution can be given by:

(5) $$\sigma_{\alpha \beta }\left(\varepsilon \right)=\frac{1}{N}\underset{i,k}{\sum }\sigma_{\alpha \beta }\left(i,k\right)\frac{\delta \left(\varepsilon -\varepsilon_{i,k}\right)}{\delta \left(\varepsilon \right)},$$

In the above relation, N denotes the number of k-points that are sampled in the BZ and $\varepsilon_{i,k}$ presents the band structure. The *k*-dependent transport tensor is read as:

(6) $$\sigma_{\alpha \beta }\left(i,k\right)=e^{2}\tau_{i,k}v_{\alpha }\left(i,k\right)v_{\beta }\left(i,k\right),$$

In this equation, i and k stand for the band index and wave vector, respectively, τ denotes the relaxation time, $v_{\alpha }\left(i,k\right)$ is the α component of the group velocities, and e is the electron charge.

By integrating the transport distribution over the energy, the electrical conductivity can then be written as a function of the temperature, *T*, and the chemical potential, μ, via the following equations:

(7) $$\sigma_{\alpha \beta }\left(T,\mu \right)=\frac{1}{\Omega }\int^{\text{​}}\sigma_{\alpha \beta }\left[-\frac{\partial f_{\mu }\left(T,\varepsilon \right)}{\partial \varepsilon }\right]d\varepsilon ,$$

(8) $$v_{\alpha \beta }\left(T,\mu \right)=\frac{1}{eT\Omega }\int^{\text{​}}\sigma_{\alpha \beta }\left(\varepsilon \right)\left(\varepsilon -\mu \right)\left[-\frac{\partial f_{\mu }\left(T,\varepsilon \right)}{\partial \varepsilon }\right]d\varepsilon ,$$

where α and β stand for the tensor indices, and Ω, μ, and f denote the volume of the unit cell, Fermi level of the carriers, and the carrier Fermi-Dirac distribution function, respectively.

Due to the complexity of carrier scattering mechanisms in the solid, the exact solution of the Boltzmann equation cannot be obtained. For this reason, the relaxation time approximation is adopted to overcome such difficulty, where the relaxation time is treated as an energy-independent constant.

## 3. Results and Discussion

### 3.1. Structural Properties

**Figure 1:**
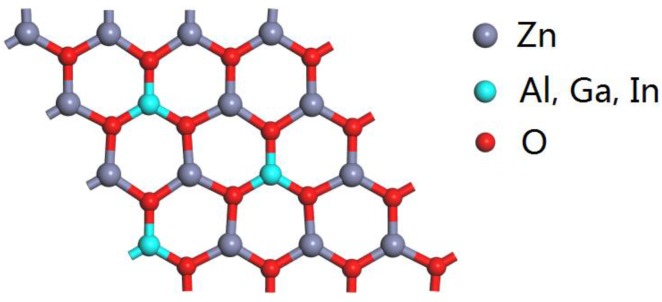
The optimized structure of Al, Ga, In doped in the ZnO monolayer.

The optimized structure of Al, Ga, and In doped in ZnO monolayers is shown in Figure 1. The atomic structure of the 2D ZnO monolayer was cut from the initial bulk ZnO (0001) plane. We have a periodic 4 × 4 × 1 supercell of the ZnO monolayer, which consists of 32 atoms with one to three Zn atoms replaced by Al, Ga, and In atoms corresponding to 6.25, 12.5, and 18.75 wt % of group Ш elements in the ZnO monolayer. In order to understand the effect of the group Ш elements on the structure of the ZnO monolayer, we have listed the structural parameters with various concentrations after the structure optimization in Table 1. It can be seen that the average length of the bonds between the Al and Ga atoms and the nearest neighbor O atoms are slightly shorter than that of the ZnO bond length. However, the average bond length between the In atoms and the nearest neighbor O atoms is around 2.1 Å, which is longer than those of Zn–O (1.9 Å). The O–Al–O bond angle and O–Ga–O bond angle is approximately 119°, and it can be known that the Al and Ga doping causes little change in the bond angle. However, the O–In–O bond angle decreased more apparently than O–Zn–O bond angle. This is due to the large difference in the atomic radii of Al, Ga, and In. The radii of Al^3+^ (0.54 Å) and Ga^3+^ (0.62Å) are less than that of Zn^2+^ (0.74 Å) and the radius of In^3+^ (0.80 Å) is larger than that of Zn^2+^.

### 3.2. Formation Energy

**Figure 2:**
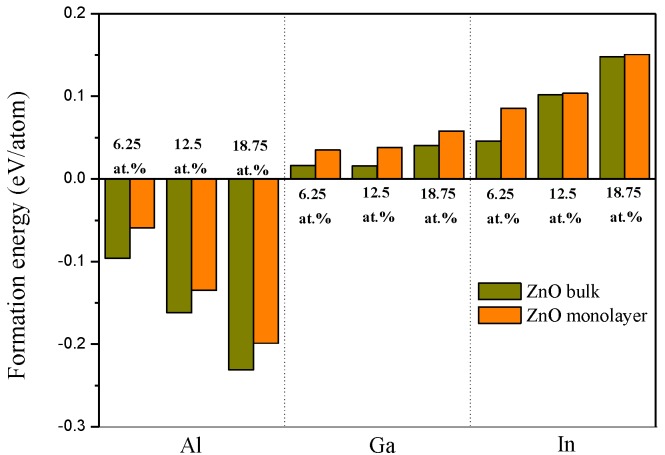
The formation energy of difference concentrations of group Ш element doped ZnO bulks and monolayers.

To examine the feasibility of the formation of group Ш element doping, we calculated the formation energies with various group Ш element concentrations in the ZnO monolayer, as shown in Figure 2. The calculation of the formation energy is used to determine the possibility of defects within the structure. The formation energy can be calculated by the following [40]:

(9) $$E_{f}=E_{defect}-\left[E_{perfect}-n\mu_{Zn}+n\mu_{M}\right],$$

where $E_{defect}$ and $E_{perfect}$ are the total energies of the supercell before and after group Ш element substitution; n is the number of substitutional group Ш atoms; $\mu_{Zn}$ and $\mu_{M}$ represent the chemical potentials of the Zn and group Ш atoms, respectively.

It is known that the thermodynamic stability is directly related to the value of formation energy. A system with smaller formation energy value is more stable. In order to describe their stability, we calculated the formation energies of the group Ш element doped ZnO bulk and monolayers in Figure 2. We have found that there is little change between the formation energies of group Ш element doped ZnO bulk and monolayers. This phenomenon signifies that group Ш elements are suitable for doping into the ZnO bulk and monolayer systems. With the Al doped ZnO monolayer, all the formation energy values are found to be negative at each Al concentration. Meanwhile, the formation energy values of Ga and In doped ZnO monolayers are around zero. In addition, the formation energies of Al doped ZnO monolayers are smaller than those observed in the case of Ga and In doped ZnO monolayers, which indicates that the doping systems can be more stable under the Al doped ZnO monolayer condition.

### 3.3. Electronic Structures

**Figure 3:**
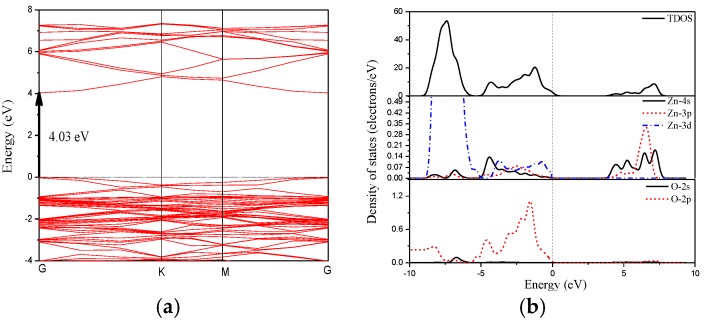
(**a**) The band structure and (**b**) density of states of the pristine ZnO monolayer.

The band structure and density of states of the pristine ZnO monolayer are closely related to the electronic structures and optical properties of group Ш element (Al, Ga, In) doped ZnO monolayers. Therefore, we first calculated the band structure and density of states after the optimized geometry structure for the pristine ZnO monolayer in Figure 3. The Fermi level indicated by a dotted line is set to zero. The conduction band minimum (CBM) and the valence band maximum (VBM) are both located at point G of the Brillouin zone, indicating that the ZnO monolayer is a direct band gap semiconductor. The valence band is mainly contributed by the Zn-3*d* and O-2*p* states. The conduction band is mainly composed of Zn-3*p* and Zn-3*d* states. The minimum gap between the VBM and CBM is 4.03 eV, which is much larger than that of the bulk material (3.37 eV) [5], due to the quantum confinement effect. It can be seen that the ZnO monolayer is a good optoelectronic material in semiconductor devices.

**Figure 4:**
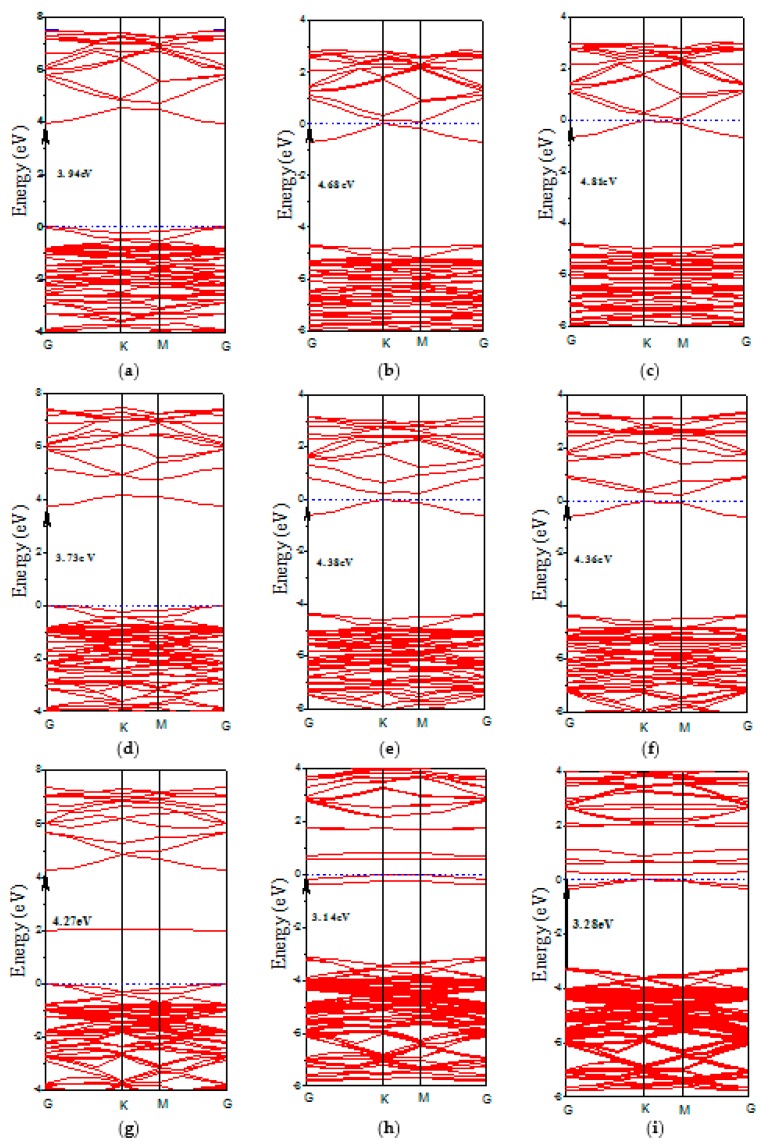
The band structures of (**a**) 6.25 wt % Al; (**b**) 12.5 wt % Al; (**c**) 18.75 wt % Al; (**d**) 6.25 wt % Ga; (**e**) 12.5 wt % Ga; (**f**) 18.75 wt % Ga; (**g**) 6.25 wt % In; (**h**) 12.5 wt % In; and (**i**) 18.75 wt % In doped ZnO monolayer.

**Figure 5:**
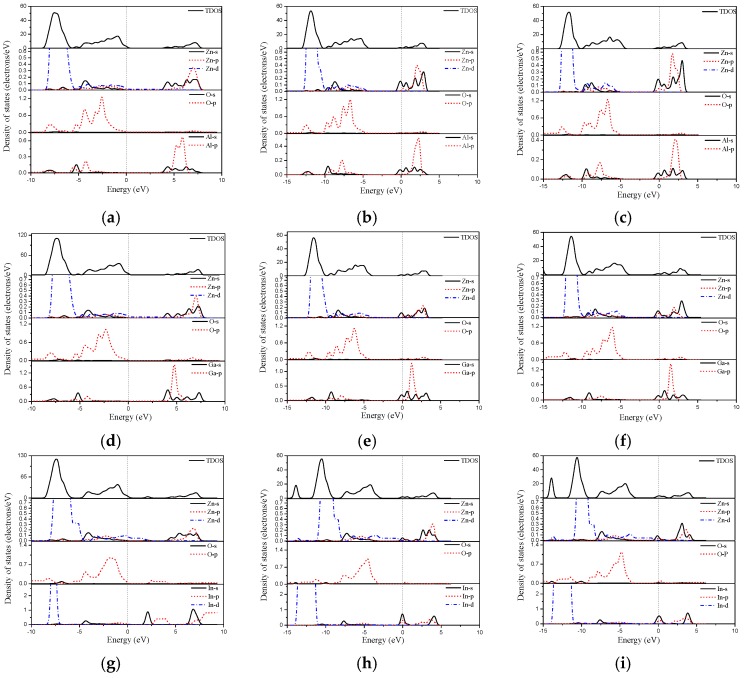
The density of states of (**a**) 6.25 wt % Al; (**b**) 12.5 wt % Al; (**c**) 18.75 wt % Al; (**d**) 6.25 wt % Ga; (**e**) 12.5 wt % Ga; (**f**) 18.75 wt % Ga; (**g**) 6.25 wt % In; (**h**) 12.5 wt % In; and (**i**) 18.75 wt % In doped ZnO monolayer.

Next, we turn to effects of the group Ш element doped ZnO monolayers on the electronic structures. The band structure and the density of states are calculated as shown in Figure 4 and Figure 5, respectively. It is noted that the Fermi level shifts upward into the conduction band when the Al doped concentration is higher than 12.5 wt %, which produces a degenerate *n*-type semiconductor. This degenerate *n*-type semiconductor is related to a pronounced Burstein-Moss-effect [41,42], which can considerably extend the apparent optical band gap. To remain, in this effect, the band gap is measured between the VBM and the Fermi level in the conduction band. This effect could be considered as the increasing conductivity in the Al, Ga, and In doped ZnO monolayers. Clearly, the Al and Ga doped ZnO monolayer is a direct-gap semiconductor with a band gap of 3.94 eV and 3.73 eV for 6.25 wt % concentration. Comparing the band structure of the pristine ZnO monolayer, we observed that the band gap of Al and Ga mono-doping ZnO monolayer decreases. However, when the Al and Ga concentrations are higher than 12.5 wt %, the band gap increases with the increase of the Al concentration. Broadening of band gap with the higher concentration Al and Ga doped ZnO monolayers may be due to the Burstein-Moss band filling effect [41,43,44]. The Fermi level shifts inside the conduction band. As the states below such shifting in the conduction band are filled, the absorption edge shifts to higher energy, resulting in a larger band gap. In the case of the In doped ZnO monolayer, the calculated band gap of the In concentration at 6.25 wt % is larger than that of the pristine ZnO monolayer. However, the band gap of the 12.5 wt % and 18.75 wt % In doped ZnO monolayers are 3.14 eV and 3.28 eV, respectively, which are smaller than that of the Al and Ga doping. The reason may also be due to the donor level corresponding to the In doped ZnO monolayer energy band that gradually widens and merges with the conduction band [45]. Therefore, the band gap became narrow by the merging of the donor and conduction bands.

The density of states was used to analyze the distribution of each related orbital associated with the constituent elements in the group Ш element doped ZnO monolayers. For the Al doped ZnO monolayer, the valence band mainly consists of the Zn-3*d* and O-2*p* states. The conduction band comes mainly from the Zn-4*s*, Zn-3*p*, and Al-3*s* states (see Figure 5a–c). In the case of the Ga doped ZnO monolayer, the conduction band is determined by the Ga-4*s* and Ga-4*p* states (Figure 5d–f), resulting in the change of the band gap width. When the Zn atoms are substituted by In atoms, we find that the valence band is mainly contributed by the In-4*d* state, as shown in Figure 5g–i. From Figure 4h,i, the occupied states around the Fermi level are mainly derived from the In-5*s* and In-5*p* states.

### 3.4. Optical Property

**Figure 6:**
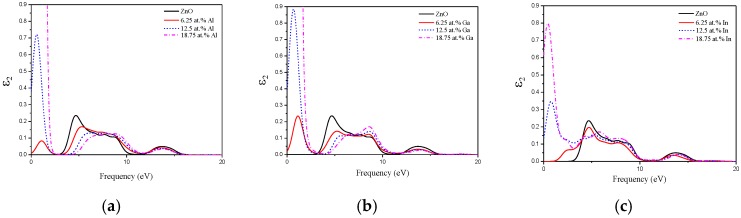
The imaginary part of the dielectric function of (**a**) Al; (**b**) Ga; and (**c**) In doped ZnO monolayer.

In this subsection, we calculate and discuss the optical properties of pristine and group Ш element (Al, Ga, and In) doped ZnO monolayers. It is well known that the complex dielectric function of a material reflects the information between the energy band structure and optical transition. So, we firstly consider the imaginary part of the dielectric function, which is plotted in Figure 6. We compare the imaginary part of the dielectric function of the difference concentrations of Al, Ga, and In doped ZnO monolayers. For the pristine ZnO monolayer, there are two main peaks at 4.69 and 13.7 eV, respectively. The main peak at 4.69 eV should mainly be caused by the optical transitions between the O 2*p* states in the highest valence band and the Zn 4*s* states in the lowest conduction band. The peak at 13.7 eV is mainly derived from the optical transition between the Zn 3*d* and O 2*s* states. With increasing concentration of the Al dopant, the main peaks in the low energy have blue shifted to a higher energy side. Meanwhile, Figure 6a shows a new peak in the low energy (<3 eV) after the Al is doped (6.25 wt % and 12.5 wt %) in the ZnO monolayer, due to the transition between the Al-3*s* donor occupied states around the Fermi level and the unoccupied Zn-4*s* and Zn-4*p* states in the conduction band. However, the new peak becomes intense at 18.75 wt % Al doped concentration. The reason may be that the high Al concentration doping makes the occupied states widen. These phenomena also occurred in the Ga and In doped ZnO monolayer system in Figure 6b,c, respectively.

**Figure 7:**
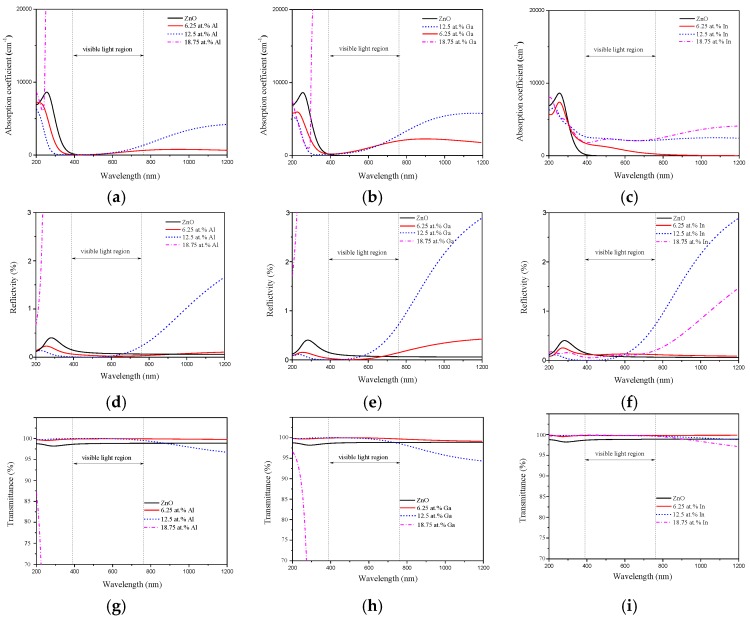
The absorption coefficient of (a) Al; (b) Ga and (c) In doped ZnO monolayer. The reflectivity of (d) Al; (e) Ga and (f) In doped ZnO monolayer. The transmittance of (g) Al; (h) Ga and (i) In doped ZnO monolayer.

The absorption coefficients of pristine and group Ш element doped ZnO monolayers have been plotted in Figure 7a–c. It is obviously seen that the pristine ZnO monolayer has a low absorption coefficient in the visible and IR regions. Compared with the pristine ZnO monolayer, the absorption edge has a clear blue-shift to a shorter wavelength region with increasing Al, Ga, and In doping concentration. However, the absorption coefficient increases from the visible to IR region when the Al and Ga concentrations are less than 12.5 wt %. Meanwhile, the absorption coefficient of Al and Ga doped ZnO monolayers at 18.75 wt % are steeply enhanced in the UV region, which means there is a significant decrease of the transmittance in the UV region when the Al and Ga doping concentration is 18.75 wt %. In the case of In doping, the absorption coefficient decreases from the UV to visible region at 6.25 wt % In concentration. However, the absorption coefficient increases in the visible and IR regions when the In concentration is higher than 12.5 wt %.

To further investigate the difference of optical properties for Al, Ga, and In doping, the reflectivity and transmittance of pristine and group Ш element doped ZnO monolayers are shown in Figure 7d–i. The reflectivity of the pristine ZnO monolayer is low in the visible and IR regions. When the Al and Ga are doped into the ZnO monolayer, it appears that there is a weaker reflectivity of the doping concentrations less than 12.5 wt % in the UV region, as shown in Figure 7d,e, respectively. However, the reflectivity of the 12.5 wt % Al doped ZnO monolayer increases obviously in the range of 600 nm–1200 nm. The reflectivity of the 6.25 wt % and 12.5 wt % Ga doped ZnO monolayers increased in the range of 500 nm–1200 nm. When the doping concentration reaches 18.75 wt %, the reflectivity of the Al and Ga doped ZnO monolayers increase steeply in the UV region. For the In doped ZnO monolayer, the reflectivity decreases with the doping concentration less than 12.5 wt % in the UV region, as shown in Figure 7f. In the visible region, the reflectivity increased slightly with 6.25 wt % doping concentration. Meanwhile, the reflectivity of the 12.5 wt % doping concentration increases from the visible to IR regions. In particular, the reflectivity increases slightly in the range of 200 nm–600 nm with the 18.75 wt % doped ZnO monolayer rather than that of the 12.5 wt %, which may be due to the decrease of the band gap value with the doping concentration at 18.75 wt %.

Furthermore, as seen from Figure 7g–i, the average transmittance of the pristine ZnO monolayer is around 97%, due to its unique planar structure. With the Al, Ga, and In doped ZnO monolayers, the average transmittance reaches up 99% in the visible and UV regions with doping concentrations less than 12.5 wt %. When the doping concentration increases to 18.75 wt %, for Al and Ga, the transmittance appears to steeply decline in the UV region. However, the In doped ZnO monolayer of 18.75 wt % concentration has a unique phenomenon, which shows the average transmittance around 99% in the visible and UV regions and above 95% in the IR region. Based on the above analyses, it is proven that the advantages of a high In concentration doped ZnO monolayer are to be the transparent materials produced when compared to the Al and Ga doped ZnO monolayers.

### 3.5. Transport Proprieties

To investigate the effect of introducing Al, Ga, or In on the electrical conductivity, we used the Boltzmann equations that are mentioned above. However, one of the major limitations of the semi-classical Boltzmann theory is the determination of the scattering rate, $\tau^{-1}$, for calculating the exact electrical conductivity. To advance in the calculations, we used the module proposed by Ong et al. [46], who have used the same method for the ZnO compound as that adopted in our calculations. The relationship of the relaxation time is given as follows:

(10) $$\tau =2.53\times 10^{-5}T^{-1}n^{-1/3},$$

In the above relationship, T denotes the temperature and n denotes the electron concentration. The temperature, here, was fixed at 300 K, which corresponded to the standard room temperature. We obtained, from this relationship, the estimated values of the relaxation times. Then we used these relaxation times to obtain the exact electrical conductivity σ as a product of (σ/τ)×τ.

**Figure 8:**
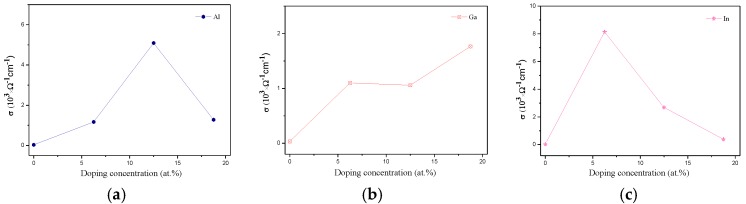
The electrical conductivity of (**a**) Al; (**b**) Ga; and (**c**) In doped ZnO monolayer.

The calculated electrical conductivity of group Ш element (Al, Ga, and In) doped ZnO monolayers as a function of the doping concentration is presented in Figure 8. The electrical conductivity is enhanced for the group Ш element doped systems compared to the pristine ZnO monolayer. The electrical conductivity decreased for Al concentrations larger than 12.5 wt %, as shown in Figure 8a. In the case of Ga, shown in Figure 8b, it is noted that the electrical conductivity increases with increasing Ga concentration. The Ga doped ZnO monolayer has a higher electrical conductivity compared to the Al and In dopants at 18.75 wt %. With the In doped ZnO monolayer, it is noted that there is a strong electrical conductivity with a high value at 6.25 wt %. However, the electrical conductivity decreases with higher concentrations of In, as shown in Figure 8c. Consequently, the 6.25 wt % In doped ZnO monolayer with high electrical conductivity and good optical properties is a promising transparent anodes material. With the Ga doped ZnO monolayer, there is about a 2% change of the Ga-O bond length. In other words, it has relatively low electron scattering. Therefore, it has a small change in the electron mobility. Moreover, the electrical conductivity increases with increasing Ga concentration. Therefore, the maximum value appears at 18.75 wt % Ga doped ZnO monolayer. In the case of Al and In doping, there is about an 8% and 11% change of the Al-O and In-O bond lengths, respectively. Because of the larger change of the structure, the electron scattering increases and the electron mobility decreases. Therefore, the electrical conductivity has fallen quickly, which forms the maximum values for the 12.5 wt % Al doped and 6.25 wt % In doped ZnO monolayer, respectively.

## 4. Conclusions

In summary, we investigated the electronic, optical, and electrical properties of 2D ZnO monolayers doped with Al, Ga, and In using the first-principles calculation in combination with the Boltzmann transport theory. We have observed that the band gap using higher than 12.5 wt % Al and Ga doping concentrations increases compared to the pristine ZnO monolayer. However, the band gap of 12.5 wt % and 18.75 wt % doping concentrations decreases compared to the pristine ZnO monolayer. The absorption edge has a clear blue-shift to a shorter wavelength region with increasing doping concentrations after Al, Ga and In doping. Meanwhile, we found that the average transmittance reaches up 99% in the visible and UV regions when ZnO monolayer is doped with Al, Ga, and In concentrations less than 12.5 wt %. In particular, it is found that the average transmittance is around 99% in the visible and UV regions and above 95% in the IR region for the 18.75 wt % In doping concentration. In addition, the electrical conductivity has increased for the Al, Ga, and In doped ZnO monolayers compared with the pristine ZnO monolayer. Furthermore, we found a higher electrical conductivity of the In doped ZnO monolayer in comparison with the ZnO monolayer doped with Al or Ga at 6.25 wt %. These features make the In doped ZnO monolayer with 6.25 wt % concentration as an excellent transparent conducting electrode for optoelectronic elements.

## Figures and Tables

**Table 1:** The bond length and bond angle of pristine and group Ш elements (Al, Ga, and In) doped in the ZnO monolayer.

Compounds	Concentrations	Bond Length (Å)		Bond Angle (°)	
		*d_Zn-O_*	*d_M-O_*	*_O-Zn-O_*	*_O-M-O_*
Pure	0%	1.910	-	120	-
Al	6.25%	1.932	1.750	119.16	119.99
	12.5%	1.944	1.751	119.05	119.98
	18.75%	1.946	1.753	119.21	119.88
Ga	6.25%	1.929	1.879	119.50	120
	12.5%	1.951	1.855	119.33	119.89
	18.75%	1.932	1.881	119.96	119.73
In	6.25%	1.921	2.136	119.97	98.34
	12.5%	1.916	2.121	118.99	110.37
	18.75%	1.926	2.116	119.97	112.96
